# Supplementary material for: Interpersonal psychotherapy delivered by nonspecialists for depression and posttraumatic stress disorder among Kenyan HIV–positive women affected by gender-based violence: Randomized controlled trial
Source: PLoS Med. 2021 Jan 11;18(1):e1003468. doi: 10.1371/journal.pmed.1003468 (PMC7799784; doi:10.1371/journal.pmed.1003468)
Supplement: S2 Table — (DOCX) [file pmed.1003468.s002.docx]

| **S2 Table: Baseline Characteristics of Completers and Non-Completers** | | | |
| --- | --- | --- | --- |
|  | **Completer**  **Mean or No. (SD or %)** | **Non-Completer**  **Mean or No. (SD or %)** | **Non-Completer /Non-Missing** |
| Average age in years | 37.9 (9.3) | 35.0 (9.3) | 88/251 |
| Depression symptoms (BDI II) | 28.9 (9.6) | 27.2 (10.8) | 88/253 |
| PTSD symptoms (PCL-C) | 55.8 (15.3) | 57.3 (17.6) | 89/254 |
| Physical intimate partner violence in past week among partnered participants (CTS)* : Yes/No | 41/70 (58.6%) | 23/35 (65.7%) | 35/251 |
| Two or more different types of lifetime trauma - e.g., crime, sexual/physical assault, disaster (THQ) | 151/ 166 (91.0%) | 82/90 (91.1%) | 90/256 |
| At least 4 different types of lifetime trauma - e.g. crime, sexual/physical assault, disaster (THQ) | 60/166 (36.1%) | 28/90 (31.1%) | 90/256 |
| Alcohol use (AUDIT >8, harmful or hazardous drinking) | 0.45 (2.23) | 0.73 (2.69) | 73/222 |
| Drug use (DAST >3, moderate and up) | 1.32 (0.95) | 1.37 (1.27) | 49/134 |
| Disability score (WHODAS)* | 31.2 (17.2) | 26.5 (18.3) | 88/252 |
| Economic productivity and absenteeism |  |  |  |
| Monthly income (USD) | 21.0 (48.3) | 12.6 (27.7) | 89/255 |
| Days in the past month partially or completely unable to work | 7.5 (9.3) | 8.2 (11.4) | 87/251 |
| Paid school fees on time in past month for those with school age children | 24/148 (16.2%) | 12/70 (17.1%) | 70/218 |
