# Supplementary material for: Interpersonal psychotherapy delivered by nonspecialists for depression and posttraumatic stress disorder among Kenyan HIV–positive women affected by gender-based violence: Randomized controlled trial
Source: PLoS Med. 2021 Jan 11;18(1):e1003468. doi: 10.1371/journal.pmed.1003468 (PMC7799784; doi:10.1371/journal.pmed.1003468)
Supplement: S1 Text — (DOCX) [file pmed.1003468.s003.docx]

**S1 Text**

**Excerpt from Mental Health and Domestic Violence Crisis Protocols: MIND Study**

**Steps taken to minimize risks to Participants and Therapists.**

Mental Health Crisis.

*Screening.* Potential participants will be advised of the study principles of overriding confidentiality for the purposes of acute mental health care in three situations: risk of self-harm, risk of harm to others and grave disability (inability to provide for one’s own food, clothing or shelter) secondary to mental illness. If the

mental status exam or MINI evaluation during study screening suggests that they need acute mental health care, they will be referred to the Family AIDS Care, Education and Services (FACES) and community resources (see below).

*IPT.* Potential participants will be informed that their mental health status will be evaluated at every study session. If their mental health status worsens during treatment such that they are at risk of self harm, harm to others or grave disability, they will be re-evaluated, referred and escorted a higher level of care/alternate care, as indicated. Potential participants will be informed that if they name an intended target of violence, the target’s name will be requested in order to advise him/her of violence protection services.

*FACES and Community Resources for Mental Health Crisis.* Resources for acute mental health care needs include evaluation and treatment with medication through FACES medical professionals, FACES mental health services, including substance abuse, individual counseling and couples counseling and area resources for psychiatric hospitalization. If the need arises for psychiatric hospitalization or mental health care needs exceeding local resources, Dr. David Bukusi will be contacted for assistance. Dr. David Bukusi is a psychiatrist on faculty at Kenyatta Hospital and has been director of HIV counseling for ten years. He is a scientific advisor on the grant funding this research. Participant will be accompanied to the referral site by study staff.

The safety of study participants will be monitored at multiple points in the study. First, their mental status will be assessed by a clinician during the screening procedure. They will be further evaluated through MINI diagnostics as well as mental and social health measures. Study therapists will be educated on recognition of mental health and GBV crisis and will monitor participants at IPT every session. The MINI and mental health measures will be repeated for participants who drop-out and at the conclusion of the study for completers. Participants who drop out of care will be contacted by telephone twice. If the effort to reach the participant is not successful, the **emergency contact**, identified by the participant at the time of study enrollment, will be contacted.

*Gender-Based Violence Crisis.*

*Screening.* If screening procedures (inclusion/exclusion criteria, MINI) suggest that the potential participant is in need of acute intervention for GBV, she will be referred to FACES and community resources including medical professionals, community elders, chiefs, counselors, church leaders, police, and a pro-bono lawyer, who have all been involved with FACES GBV interventions. Funds will be allocated to assist with immediate transport for urgent GBV needs. Participant will be accompanied to the referral site by study staff.

*IPT.* Potential participants will be informed that if therapy sessions suggest that they need urgent intervention related to GBV, the research team will attempt to obtain further information and provide referrals through FACES and community resources. Potential participants will be informed that if they name an intended target of violence, the target’s name will be requested in order to advise him/her of violence protection

services.

*FACES and Community Resources for GBV Crisis.* Existing resource persons include medical professionals, community elders, chiefs, counselors, church leaders, police, and a pro-bono lawyer, who have all been involved with FACES GBV interventions. Funds will be allocated to assist with immediate transport for urgent

GBV needs. Participant will be accompanied to the referral site by study staff. The safety of study participants will be monitored at multiple points in the study. First, their mental status will be assessed by a clinician during the screening procedure. The will be further evaluated through MINI diagnostics as well as mental and social health measures. Study therapists will be educated on recognition of mental health and GBV crisis and will monitor participants at IPT every session. The MINI and mental health measures will be repeated for participants who drop-out and at the conclusion of the study for completers.

Safety of Therapists

Care of therapists and prevention of vicarious traumatization will be central to this study. The concept of vicarious traumatization will be introduced, with role playing during IPT didactics. Coping skills will be reviewed and practiced. One of the primary components of supervision will be the monitoring of the emotional health of the therapist, given the intensity of therapy with depressed and traumatized participants. In addition to this focused, confidential supervision, peer support groups will be developed where therapists can meet informally to socialize and relax with one another. If therapists are judged to be having emotional difficulty, they will be assessed and referred for mental health care services. If the work appears to interfere with their ability to safely complete the study work, they will be given a leave of absence from the study.
