# Supplementary material for: Interpersonal psychotherapy delivered by nonspecialists for depression and posttraumatic stress disorder among Kenyan HIV–positive women affected by gender-based violence: Randomized controlled trial
Source: PLoS Med. 2021 Jan 11;18(1):e1003468. doi: 10.1371/journal.pmed.1003468 (PMC7799784; doi:10.1371/journal.pmed.1003468)
Supplement: Attachment — Submitted filename: Response to Reviewers and Editors.docx [file pmed.1003468.s004.docx]

**Point-by-Point Response to Editors and Reviewers**

**Comments from Editors.**

*To your title, please add a study descriptor following a colon, e.g., "...: a randomized controlled trial".*
We changed our title to: “Interpersonal Psychotherapy Delivered by Non-Specialists for Depression and Posttraumatic Stress Disorder Among Kenyan HIV-Positive Women Affected by Gender-based Violence: Randomized Controlled Trial.”

*Please combine the "Methods" and "findings" section of your abstract, in accordance with journal style, and add a new final sentence to the new combined subsection quoting 2-3 of the study's main limitations*.

We combined the Methods and Findings sections of the abstract and added a new final sentence quoting the study’s main limitations (page 2):

Limitations: (1) 35.2% attrition inclusive of follow-up assessments; (2) Generalizability to populations not in HIV care; (3) Data not collected on TAU resources accessed.

*After the abstract, we will need to ask you to add a new and accessible "author summary" section in non-identical prose. You may find it helpful to consult one or two recent research papers published in PLOS Medicine to get a sense of the preferred style.*

Author Summary:

**Why was the study done?**

**>** HIV-positive women in sub-Saharan Africa experience high levels of Gender-Based Violence (GBV), leading to an extraordinarily high prevalence of mental disorders, particularly depression and Posttraumatic Stress Disorder (PTSD).

**>** Despite knowledge that evidence-based psychotherapy for depression and PTSD can be delivered by local non-specialists in East Africa with high efficacy, little data exists on scalable treatment models for HIV-positive women affected by GBV in the region.

**What did the researchers do and find?**

**>** We partnered with a large HIV clinic in western Kenya to conduct a randomized controlled trial of Interpersonal Psychotherapy (IPT) versus Wait List-Treatment As Usual (TAU). Participants were 256 women enrolled in HIV care and affected by GBV who met criteria for Major Depressive Disorder and PTSD (primary outcomes).

**>** We used a scalable intervention in which local non-specialists (no prior mental health training required) were trained to deliver IPT inside an HIV clinic, working closely with HIV clinic staff and providers.

**>** The overall design was an Effectiveness-Implementation Hybrid, which involves data collection on both effectiveness and implementation outcomes in a single study, with the goal of efficiently producing data for service scale up.

**>** At the conclusion of treatment, those who received IPT had significant reduction in the odds of MDD (75%), PTSD (65%) and combined MDD-PTSD (64%) compared to Wait List+TAU controls. Wait List+TAU participants experienced similar remission after they received IPT treatment and gains were maintained across follow-up (6months for IPT+TAU and 3 months for Wait List+TAU).

**>** Secondary Findings:

**>** IPT participants had a greater reduction of disability (37.5%), and a trend toward reduced work absenteeism compared to controls.

**>** For IPT receipts with intimate partners, there was a 71% decrease in physical intimate partner violence, significantly greater than controls.

**What do these findings mean?**

**>** This study suggests that Interpersonal Psychotherapy can be delivered in a scalable manner, including administration by non-specialists, housed within existing HIV clinics.

**>** Delivering IPT to HIV-positive women affected by GBV using clinic-integrated non-specialists achieves substantial remission of MDD and PTSD sustained over six-month follow-up, with attendant reductions in disability and a trend toward improved work attendance, as well as substantial reductions in physical violence by intimate partners.

*To your methods section, please add some additional detail on study ethics approvals, e.g., date of approval and application numbers. Please state whether consent was written or verbal, and whether informed.*

These details were added (page 6):
The UCSF Committee on Human Research (IRB# 13-11765) approved the protocol on March 17, 2014) and the Kenya Medical Research Institute (KEMRI) Scientific Ethical Review Unit (SSC Protocol No. 2753) approved the protocol on May 26, 2014. Secondary to literacy rates in the region, written or thumbprint informed consent was used for all participants. Prospective participants were also assessed for their understanding of each point of the informed consent—they were asked to repeat each point of the informed consent in their own words (three efforts allowed), and the point was initialed by the research staff.

*Please remove the results of baseline statistical tests from table* 1.

Baseline statistical tests were removed from Table 1.

*Please remove the statement about data sharing from the end of the main text - this information will appear in the article metadata in the event of publication, via entries in the submission form. Please also consult our policy on data sharing - a non-author contact for inquiries about access to study data will be needed (https://urldefense.proofpoint.com/v2/url?u=https-3A__journals.plos.org_plosmedicine_s_data-2Davailability&d=DwIGaQ&c=iORugZls2LlYyCAZRB3XLg&r=kVvsBRhJCDWofb155NoTYQkoE9tPvj5RZdl_rcoG-dg&m=KnzGggkL-eRBoCf1Oys13RihqQFCtncMC-GSz9osaJ8&s=ETNF6e7aNr6F3LXBm-dTI-__DclWVdjBV1m3o9F4zIs&e= ).*

Statement was removed.

*Please make that "sub-Saharan Africa" throughout.*

Change was made.

*Please state exact p values or "p<0.001".*

P values were corrected to 2 digits of precision, with 3 digits given if p value is between .01 and .001 and smaller as p<.001.

*Throughout the text, please adapt reference call-outs to the following style: "... general population [1,2]." (i.e., no spaces within the square brackets).*

Corrected.

*In your reference list, please convert italics into plain text. Where appropriate, 6 author names should be listed rather than 3, followed by "et al.".*

Corrected.

*Please add a completed CONSORT checklist as a supplementary document, referred to in your methods section (e.g., "See S1_CONSORT_Checklist"). In the checklist, individual items should be referred to by section (e.g., "Methods") and paragraph number rather than by line or page numbers, as the latter generally change in the event of publication.*

This was completed and uploaded in the electronic submission.

**Comments from the Reviewers:**

**Reviewer #1.**

This review relates to manuscript PMEDICINE-D-20-02128 about Effectiveness of Interpersonal Psychotherapy Delivered by Non-Specialists for Depression and Posttraumatic Stress Disorder Among Kenyan HIV-Positive Women Affected by Gender-based Violence.
I have listed my comments, major or minor, below.

Major comments:

*I believe the flow of the manuscript could be improved. In particular, a lot of information included in the "Study design" section consists in background information. These two sections need to be reorganized.*

We moved our discussion of effectiveness-implementation studies and our comparator condition from the “Study Design” to our “Background” and “Objective” sections (pages 3, 4).

*I am concerned about potential multiplicity issues due to the number of primary outcomes. It is also unclear which outcomes should be considered primary. The outcome section list 4 'primary outcomes'. The authors also write that they used both continuous and dichotomous versions of MDD and PTSD. In other places only MDD and PTSD are listed as the two primary outcomes. Please clarify as well as discuss the potential multiplicity issue including how one might control the risk of false-positive results in light of the number of tests conducted.*

We clarified that our primary outcomes are MDD and PTSD diagnoses (binary outcome) on the Mini International Neuropsychiatric Interview (page 8). Continuous measures were used as secondary outcomes to countercheck via convergent validity. The continuous measures were moved to the secondary outcome section and this point was clarified.

*Randomization: A colored Excel list was used as the randomisation sequence. If the study coordinator performing the randomisation has access to the Excel file, s/he was able to identify upcoming allocations. This lack of concealment comes with a potential high-risk of bias. Please clarify whether future allocations were concealed and if measures were in place to ensure no preferential allocation (e.g. skipping an allocation to 'force' a participant to be allocated to a specific group).*

Thank you for the helpful comment. We clarified the randomization procedures (page 9):

The study coordinator generated a random sequence of two colors in Excel corresponding to IPT+TAU and Wait List+TAU in blocks of 10. After completion of baseline measures, the study coordinator assigned participants to the next listed random color block and its corresponding treatment assignment. While the next assignment was visible to the study coordinator, no skipping was allowed and order of participant arrival was not altered.

*Given the substantial amount of participants with missing outcome data, I would suggest some sensitivity analyses to assess the robustness of the findings under various assumptions about the missing data. For example, one could use a range of simple imputations to determine the tipping point, or the point at which the results are no longer consistent with the main (non-imputed) findings. Please refer to the following paper for example:* [*https://urldefense.proofpoint.com/v2/url?u=https-3A__pubmed.ncbi.nlm.nih.gov_12483769_&d=DwIGaQ&c=iORugZls2LlYyCAZRB3XLg&r=kVvsBRhJCDWofb155NoTYQkoE9tPvj5RZdl_rcoG-dg&m=KnzGggkL-eRBoCf1Oys13RihqQFCtncMC-GSz9osaJ8&s=BSa6ye2oUzHmIq-o-C9TnzD9ppOXKDHt1ngV__e8jIw&e=*](https://urldefense.proofpoint.com/v2/url?u=https-3A__pubmed.ncbi.nlm.nih.gov_12483769_&d=DwIGaQ&c=iORugZls2LlYyCAZRB3XLg&r=kVvsBRhJCDWofb155NoTYQkoE9tPvj5RZdl_rcoG-dg&m=KnzGggkL-eRBoCf1Oys13RihqQFCtncMC-GSz9osaJ8&s=BSa6ye2oUzHmIq-o-C9TnzD9ppOXKDHt1ngV__e8jIw&e=)

We conducted sensitivity analyses on missing data and included the below text in the manuscript (page 17):

At the key time point (3 months) there were missing data on the primary outcomes for 1 completer in the Wait List+TAU group and 13 in the IPT+TAU group. We conducted a sensitivity analysis (1) by checking all possible allocations of the missing data (so 2*14=28 possibilities in all) to have or not have remission for that outcome. For each allocation we recalculated the p-value. For the outcome of MDD, statistical significance was very robust to the missing data with only a single allocation not being statistically significant (allocating the missing Wait List+TAU to remission and none of the 13 in the IPT+TAU group to remission); even that allocation had a borderline p-value of 0.068. The analyses for PTSD and the combined MDD and PTSD outcomes were somewhat less robust. Using the observed rates we would expect about a 50% chance of the Wait List+TAU missing data to be a remission and between 10 and 11 of the IPT+TAU group to achieve remission. If 8 or fewer of the IPT+TAU with missing data were allocated to be remitters then statistical significance was lost.

*I would strongly encourage all pre-specified study outcomes to be reported in this main manuscript. I would expect to see at least all primary and secondary outcomes as well as details of the intervention delivery and adherence. I believe a decision not to report all outcomes should be reported by strong justification.*

Thank you for this important comment. We thought through this observation carefully and elected to add our full complement of mental health outcomes, including the results of IPT impact on intimate partner violence, and our quantitative implementation outcomes relevant to the interventions (feasibility and fidelity). We also added a manuscript section that lists all the study outcomes and identifies those that were and were not included in the manuscript, with accompanying rationale (page 5):

As detailed in the published study protocol [2], the effectiveness aspect of the study included a mental health clinical core, neurocognitive testing, HIV treatment adherence, and HIV viral load on a participant sub-sample, while the implementation domains were assessed using qualitative analyses of acceptability and appropriateness, as well as quantitative measures of feasibility, treatment fidelity and economic productivity and treatment cost-benefit analyses. Given the breadth of outcome data and space limitations, we are not able to report all the study’s findings with adequate discussion here. Therefore, we focus on the mental health clinical core, which included the study’s primary outcomes and key secondary outcomes, as well as implementation outcomes with direct relevance to the intervention—feasibility and fidelity.

*Please attach the study protocol and/or statistical analysis plan as a supplement. The statistical analysis plan should clearly indicate which analyses were pre-specified vs post-hoc, i.e. decided after seeing unblinded results.*

We were not able to attach the study protocol as a supplement because it was previously published, but we referenced it more clearly (see above response), page 5.

Minor comments:
 *Abstract: please indicate the specific setting (e.g. city + country)*

This was added.

*Abstract: please indicate the timing corresponding to the primary outcome results e.g. 3 months*

This was added.

*Abstract: please correct typo in confidence interval for OR (0.0.60)*

Corrected.

*Objective section: please clarify what a "Type I trial" is.*

Effectiveness-Implementation Hybrid designs, including the Type I variant was clarified (page 5):

Effectiveness-implementation hybrid studies have developed over the past decade to collect both clinical and implementation data simultaneously. The goal of these designs is to speed translation of positive findings toward scale up and benefit to public health by collecting implementation data earlier in the pipeline than traditionally done, allowing for earlier recognition of scalable (and not scalable) interventions.[2,3] “Type I” Effectiveness-Implementation hybrid designs (as with this study) emphasize effectiveness outcomes, while collecting initial data on implementation parameters.[4]

*Objective section: The authors write that "The diversity of secondary study outcomes exceeds the scope of a single manuscript". It may indeed be difficult to report all outcomes in a single manuscript; however, I would like to see a complete list of all pre-specified study outcomes listed accompanied by a clear indication of which ones are reported in the current manuscript. According to the ClinicalTrial.gov record, there is only a limited number of outcomes measures in which case I believe all should be reported in the current paper.*

Thank you for this important point. As noted above, we added as section describing our rationale and a table listing all pre-specified outcomes, noting which are reported in this manuscript (Table 1, page 6).

*Please consider adding details of the intervention including its various components. This could be included as a supplement.*

Page 7: We added a reference to our published case history of IPT treatment in the MIND study, including a description of IPT content and delivery in this study population.[5]

*Methods: consider moving the sub-section labelled "HIV clinic setting" to the background section.*

We moved our discussion of the HIV clinic setting to the background section (page 4).

*Methods, study design: the first paragraph repeats the objective sub-section. Please remove the repeated information from the objective sub-section and consolidate. I would suggest that the second paragraph (starting with "Wait List-TAU comparators for psychotherapy…") be moved to the background section.*

Thank you for picking these up. We removed the repetitive sentence from the methods section. We moved the Wait List-TAU comparator discussion to the study background section.

*Sample size: I note that the sample size was calculated to detect an effect size (Cohen's d) of 0.8 which is very large. Apart from the earlier study (reference 41) used as a reference, are there other sources supporting such a large effect?*

We elaborated on the literature for mental health delivery by non-specialists in Low and Middle Income Countries (LMICs) and consistent findings across teams and settings of quite strong effect sizes, much higher than typical in High Income Countries (page 9):

In our prior work using a similar IPT treatment for a traumatized, low resource population, we observed a Cohen's d effect size of .79 for depression symptom improvement.[5] While this effect size is large, it is consistent with other studies of evidence-based psychotherapy in LMICs [6–8]. The depression Cohen's d indicated need for a sample size of ≥50 for a 2 group design with power of 0.8 and probability level of .05. Recognizing the migration patterns of the study population and the study requirement to complete four assessments across 36 weeks, with substantial time investment at each assessment, we allowed for approximately 20% attrition at each assessment point and selected a sample size of 100. Because we collected venous blood samples for HIV viral load testing (data in preparation) in a subgroup of participants, which required multiple venipunctures, and fear of venipuncture can raise attrition, we increased our sample size by 50% per assessment point, estimating the need to screen approximately 380 participants to reach our recruitment goal.

*Multivariate statistics: please add details about the main model. In particular, please clarify the multilevel components including what were the level(s) and how the structure (correlations) were accounted for. Please explicitly list all fixed and random effects included in the model. Was the baseline value of the outcome (MDD or PTSD) included as a covariate?*

We used fixed effects for time (categorical), intervention group and the group by time interaction. We included a random intercept for participant; this induces an exchangeable correlation structure on the logit scale (for the binary outcomes) or on the original scale (for the numeric outcomes). For the primary outcomes, all participants started at 100% so there was no advantage in adjusting. For the numeric outcomes, in place of adjustment we included the baseline values as an outcome in the analysis. The Methods section has been amended as follows (page 10):

The primary outcomes (binary), MDD and PTSD, were separately analyzed using intention-to-treat methods with multilevel mixed-effects logistic regression to assess the differences between treatment groups over time. We included a random effect (intercept) for participant and fixed effects of treatment group, time (as a categorical variable) and the interaction between time and treatment group. Both groups started with all participants diagnosed with both MDD and PTSD on the baseline MINI v5 and so that time point was left out of the analyses. The main group comparison of interest was at week 12, when the IPT+TAU group had completed IPT and the Wait List+TAU group had not yet begun IPT.

Most of our secondary outcomes were analyzed using linear mixed models (with the same random and fixed effects as above), including BDI, PCL and WHODAS. Physical Intimate Partner Violence (IPV) was derived from the CTS (above) and coded as a binary variable, because “no violence” is a goal of clinical care. Physical IPV was assessed in the subgroup of partnered participants using multilevel mixed-effects logistic regression to assess the interaction between treatment group for repeated measures over time. We included a random effect (intercept) for participant and fixed effects of treatment group, time (as a categorical variable) and the interaction between time and treatment group.

*Please indicate the statistical software(s) used for the analyses*

This information was added (page 11):
All analyses were conducted using Stata 15 [9]

*Please include a table comparing baseline characteristics of completers vs non-completers in the online supplement.*
Table was included in the supplement.

*When reporting the effect of the intervention on outcomes, the authors talk about significant group by time interaction at specific timepoints. For example: "the only significant group by time interaction for MDD was at month three". In this case, it is not the interaction that is significant but the difference between intervention arms at a specific timepoint. The interaction measures the overall consistency of the intervention effect across the 3 timepoints. Please amend accordingly.*

We clarified our results reporting across the manuscript to indicate that we are not reporting consistency across time points, but rather that the only time point of key interest was at month 3, when the two groups differed in regards to their receipt of IPT (IPT+TAU group had received IPT and Wait List-TAU had not yet received IPT).

*Please describe adherence to the intervention. For example, duration of the sessions, proportions attending each session, etc.*

We added a manuscript section on our implementation outcomes, including fidelity (IPT adherence). These measures were part of our pre-specified Implementation outcomes (page 11):

**Fidelity.**

IPT adherence was scored using a 10- point Likert scale assessing adherence in each of the three IPT phases. Sessions were considered adherent to the IPT protocol if they averaged a score of 5 or higher and did not employ off-protocol interventions.[2] Sessions were required to last one hour. Longer sessions were discouraged and shorter sessions were considered off-protocol. While audio recording of the sessions were made, due to unexpected expenses, the budget did not allow for a random 20% of sessions to be independently evaluated, as planned.[2] In place of this, we initiated local peer IPT supervision as an adjunct to and countercheck of adherence (see “interventions” for details).

*When reporting secondary outcomes (disability and absenteeism), the authors mention "controlling for the effect of time..". Please clarify what this means. Is this using a different model than for the primary outcome(s)?*

We used the same analysis for disability and absenteeism as we did for the primary outcomes. We eliminated the phrase “controlling for the effect of time” in order to avoid confusion (page 18).


**Reviewer #2:**

*Dear Authors
Thank you for the opportunity to review this manuscript reporting individually randomized 2-arm trial of an interpersonal psychotherapy for HIV-GBV+ women with co-morbid depression and PTSD in a low resource setting. Areas for improvement include explicitly reporting data as required by the CONSORT statement. For example, explicitly reporting the randomization ratio. I suggest the authors to revise the manuscript against the CONSORT checklist, add missing information and submit revised manuscript alongside completed CONSORT checklist.*

We added a CONSORT checklist and any missing items.

*I have never come across "a hybrid effectiveness-implementation design type I weighted toward evaluation of effectiveness outcomes". To my view, this study is a standard individually randomized 2-arm trial. If the authors insist on the hybrid design, this choice should be justified and supported by relevant references.
The follow up period of 6 months for the intervention arm and 3 months for the control arm is not enough to prove the sustainability of the intervention effect. Authors' claims about sustainable effect should be toned down.*

The reviewer is correct; this is a randomized controlled trial. The selection and organization of outcome measures also qualify it as an effectiveness-implementation hybrid study, given the explicit clinical and implementation domains covered by the outcomes measures. We clarified this (including references) in the ”Objective” section of the manuscript (page 6):

The study aimed to test a sustainable, scalable model for treating comorbid MDD and PTSD among GBV-affected HIV-positive women. We conducted a randomized, effectiveness-implementation type I trial of IPT plus TAU delivered by non-specialists in an HIV clinic compared to Wait List+TAU for care-engaged HIV-positive women (Figure 1 and study design, below). Effectiveness-Implementation Hybrid studies have developed over the past decade to collect both clinical and implementation data simultaneously. The goal of these designs is to speed translation of positive findings toward scale up and benefit to public health by collecting implementation data earlier in the pipeline than traditionally done, allowing for faster recognition of scalable (and not scalable) interventions.[3,5] “Type I” Effectiveness-Implementation Hybrid designs (as with this study) emphasize effectiveness outcomes, while collecting initial data on implementation parameters.[4]

*p. 13. It seems that some text slipped during formatting. Paragraph under Table 1 should be under Baseline Univariate Statistics not under Non-Completer Analyses.*

Thank you for noting this. Corrected.

**Reviewer #3:**

*This study is an important contribution to the literature as it describes the results of a fully-powered RCT effectiveness-implementation hybrid design to test the effectiveness of IPT in addressing comorbid MDD and PTSD in GBV-affected HIV-positive women in Kenya, which is a highly understudied population. Moreover, the providers were non-specialists and treatment was delivered in HIV clinics, which makes it a novel and potentially more sustainable and scale-able intervention.*

*Overall the manuscript is quite strong. I have listed comments below that may further strengthen this article.*
Thank you.

*The authors chose to focus exclusively on improvement of mental health outcomes in the manuscript, but the study is framed specifically as an "effectiveness-implementation" trial. It would have been helpful to learn more about the implementation outcomes of the trial, in addition to changes in the mental health outcomes. For example, information on feasibility, fidelity, acceptability, satisfaction, and barriers and facilitators to treatment delivery would have been useful in understanding and interpreting the outcomes*.

Thank you. We added a “Table 1” that describes all of our study outcomes and the ones reported in the manuscript with rationale. We added reporting of quantitative implementation outcomes (feasibility and fidelity) (Table 1, page , pages 21, 23 and 24).

*Similarly, although the intervention specifically targets HIV+ women who have experienced GBV, changes in GBV and HIV adherence/viral load were not reported. The authors note that they have space limitations, but it would be helpful for the reader to have these outcomes reported.*

We added our findings on intimate partner violence (abstract, summary and pages 21, 23). We added a “Table 1” detailing all study outcomes and our rationale for which outcomes we report (page 7). Given budget and timing limitations we were only able to obtain complete HIV outcomes (antiretroviral therapy adherence [ART] and viral load) on a sub-group of participants. Analysis of this sub-set focuses primarily on those who were viremic at baseline (an even smaller group) and requires detailed analyses and discussion to address potential bias via regression to the mean. Given the differences in study group and analytic methods, we felt that presentation and discussion of those results could not be incorporated with the current manuscript.

*PARTICIPANTS: The authors note that participants were diagnosed using the MINI 5.0. Who conducted the assessments? What was their training?*

The PI (S. Meffert) is a psychiatrist and is trained in administration of the MINI. She trained the clinical evaluators who applied this measure for the study. The MINI can be applied by trained lay personnel (https://harmresearch.org/index.php/mini-international-neuropsychiatric-interview-mini/).

*Were women currently experiencing GBV, and if so, how was risk of harm addressed during the study?*

The team was alert to the many risks this group of women could encounter, including increased domestic violence, suicidality and homicidaility, among others. We were also aware of the risk of vicarious trauma to non-specialist therapists. An excerpt from our mental health crises plan is included below and added it to the supplementary materials.

**Steps taken to minimize risks to Participants and Therapists.**

Mental Health Crisis.

*Screening.* Potential participants will be advised of the study principles of overriding confidentiality for the purposes of acute mental health care in three situations: risk of self-harm, risk of harm to others and grave disability (inability to provide for one’s own food, clothing or shelter) secondary to mental illness. If the

mental status exam or MINI evaluation during study screening suggests that they need acute mental health care, they will be referred to the Family AIDS Care, Education and Services (FACES) and community resources (see below).

*IPT.* Potential participants will be informed that their mental health status will be evaluated at every study session. If their mental health status worsens during treatment such that they are at risk of self harm, harm to others or grave disability, they will be re-evaluated, referred and escorted a higher level of care/alternate care, as indicated. Potential participants will be informed that if they name an intended target of violence, the target’s name will be requested in order to advise him/her of violence protection services.

*FACES and Community Resources for Mental Health Crisis.* Resources for acute mental health care needs include evaluation and treatment with medication through FACES medical professionals, FACES mental health services, including substance abuse, individual counseling and couples counseling and area resources for psychiatric hospitalization. If the need arises for psychiatric hospitalization or mental health care needs exceeding local resources, Dr. David Bukusi will be contacted for assistance. Dr. David Bukusi is a psychiatrist on faculty at Kenyatta Hospital and has been director of HIV counseling for ten years. He is a scientific advisor on the grant funding this research. Participant will be accompanied to the referral site by study staff.

The safety of study participants will be monitored at multiple points in the study. First, their mental status will be assessed by a clinician during the screening procedure. They will be further evaluated through MINI diagnostics as well as mental and social health measures. Study therapists will be educated on recognition of mental health and GBV crisis and will monitor participants at IPT every session. The MINI and mental health measures will be repeated for participants who drop-out and at the conclusion of the study for completers. Participants who drop out of care will be contacted by telephone twice. If the effort to reach the participant is not successful, the **emergency contact**, identified by the participant at the time of study enrollment, will be contacted.

*Gender-Based Violence Crisis.*

*Screening.* If screening procedures (inclusion/exclusion criteria, MINI) suggest that the potential participant is in need of acute intervention for GBV, she will be referred to FACES and community resources including medical professionals, community elders, chiefs, counselors, church leaders, police, and a pro-bono lawyer, who have all been involved with FACES GBV interventions. Funds will be allocated to assist with immediate transport for urgent GBV needs. Participant will be accompanied to the referral site by study staff.

*IPT.* Potential participants will be informed that if therapy sessions suggest that they need urgent intervention related to GBV, the research team will attempt to obtain further information and provide referrals through FACES and community resources. Potential participants will be informed that if they name an intended target of violence, the target’s name will be requested in order to advise him/her of violence protection

services.

*FACES and Community Resources for GBV Crisis.* Existing resource persons include medical professionals, community elders, chiefs, counselors, church leaders, police, and a pro-bono lawyer, who have all been involved with FACES GBV interventions. Funds will be allocated to assist with immediate transport for urgent

GBV needs. Participant will be accompanied to the referral site by study staff. The safety of study participants will be monitored at multiple points in the study. First, their mental status will be assessed by a clinician during the screening procedure. The will be further evaluated through MINI diagnostics as well as mental and social health measures. Study therapists will be educated on recognition of mental health and GBV crisis and will monitor participants at IPT every session. The MINI and mental health measures will be repeated for participants who drop-out and at the conclusion of the study for completers.

Safety of Therapists

Care of therapists and prevention of vicarious traumatization will be central to this study. The concept of vicarious traumatization will be introduced, with role playing during IPT didactics. Coping skills will be reviewed and practiced. One of the primary components of supervision will be the monitoring of the emotional health of the therapist, given the intensity of therapy with depressed and traumatized participants. In addition to this focused, confidential supervision, peer support groups will be developed where therapists can meet informally to socialize and relax with one another. If therapists are judged to be having emotional difficulty, they will be assessed and referred for mental health care services. If the work appears to interfere with their ability to safely complete the study work, they will be given a leave of absence from the study.

*Additionally, how were exclusion criteria of severe thought and mood disorder and current drug and alcohol dependence assessed and addressed? By whom?*

This was clarified in the manuscript (page 6):

Cognitive dysfunction and severe thought disorder were screened by clinical evaluators using orientation questions. Participants were asked to state their name, location and the approximate date, as well as their reason for meeting with the team. If they could not answer these questions, they were referred for medical evaluation. Current alcohol and drug dependence were assessed by the clinical evaluators using the AUDIT and DAST, with cut offs of 8 and 3, respectively, indicating harmful/hazardous alcohol use/moderate drug use (Table 2). All participants were screened for suicidality and if they screened positive, they were transported using study funds and with study staff escort to the local inpatient psychiatry unit for evaluation.

*Did the participants receive any compensation for assessments, treatment visits, or transportation costs?*

We compensated study participants for their travel costs and this was added to the manuscript methods section (page 7):

*Compensation.* Many of the women in this study were economically disempowered and lived in rural areas, making weekly transport to the HIV clinic a significant financial challenge. Therefore, participants received 300ksh (3usd) to cover transport for their weekly IPT sessions and study assessments.

We added text on this compensation to the discussion section of the manuscript (page 18):

As described, the study provided 300ksh (~3USD) to participants to cover travel costs, given the distance they traveled for weekly IPT sessions. In our view, this is not a limitation of the study, but rather an important consideration for implementation of psychotherapy in LMICs. We look forward to calculating these transport costs as part of the IPT intervention with our forthcoming cost analyses.

*RECRUITMENT. The authors state that "Patients were recruited through FACES HIV provider referral and self-referral following informational talks to FACES patients in the HIV clinic check-in and waiting areas. Consecutive HIV-positive women were consented and screened for inclusion from May 2015-July 2016." How many women were referred by providers out of the total case load? What criteria did providers use to determine whether referral was appropriate? How many women were given the informational talks? How many self-referred? Of the provider vs. self-referrals, how many met inclusion and exclusion criteria?*

Unfortunately, we did not collect data that would allow us to address all of these points. However, we were able to use our files to calculate estimates of self-referral versus HIV provider referral (page 10):

Approximately 90% of participants self-referred after listening to a “health talk” at the clinic describing the study and roughly 10% were referred by HIV clinic staff and providers who also heard the health talk.

*How many prospective IPT therapists applied and how many went on to meet the requirements of being a therapist?*

We added a manuscript results section describing IPT therapist training outcomes (page 11):

We began the training sessions with 21 prospective IPT therapists. Ten of these joined the study after successfully completing the required didactic training and IPT practice cases (with average IPT adherence score of “9” or higher).

*RESULTS. Participants in the intervention group had significantly greater symptom and functional improvement compared to the TAU group. However, the TAU group also showed improvements on depression, PTSD, and functional impairment outcomes. The improvement is so marked that participants in TAU had PCL scores that were below the clinical cut-point before they received the intervention. This improvement in symptom change is particularly notable given that all participants met MINI criteria for MDD and PTSD at baseline. What do you think may be accounting for such improvement in the TAU condition?*

Excellent point. We have thought carefully about this and have included possibilities in our discussion (page 18):

This study found IPT highly effective for comorbid MDD and PTSD among HIV-positive women affected by GBV, with high rates of remission, and clinically significant decrease of symptom severity for both disorders, with all improvements sustained over six month follow up. Interestingly, the Wait List-TAU group decreased in MDD, PTSD and corresponding symptoms between baseline and month 3, prior to receipt of IPT. This could be due to a number of reasons related to the study’s setting and population: (1) for many of these women, this study was the first time they had ever been asked about their emotions and experiences with GBV by a sympathetic and respectful person—this, alone, could have been cathartic; (2) as described above, almost none of these women had ever received any formal mental health care—the hope that was instilled by their enrollment in the study and upcoming IPT could have improved their mental health even before treatment started. In secondary outcomes analyses, IPT decreased health-related disability and improved employment outcomes for IPT+TAU participants relative to Wait List+TAU controls. IPT was also associated with decrease in violent intimate partner violence for the study’s subset of partnered participants. In regards to implementation outcomes, IPT adherence was strong and local peer IPT supervision group was feasible. Despite fairly rigorous pre-specified feasibility benchmarks, the study met two of the four, missing the screening/enrollment window slightly and experiencing higher than expected attrition (see limitations). Overall, the study showed that IPT effectiveness is strong in a “real-world” setting with delivery by local non-specialists and integrated within existing HIV clinic operations.

*SMALL Typos:*
*At each assessment visit, BID and PCL were analyzed as numerical scores". Should be BDI*

Corrected.

*At month three, LFTU was higher in the IPT+TAU group than the Wait List+TAU (27.6% (34/123) vs. 12% (16/133), p<0.01)." LFTU is not defined.*

Corrected.

**REFERENCES**

1.	Hollis S. A graphical sensitivity analysis for clinical trials with non-ignorable missing binary outcome. Stat Med. 2002 Dec 30;21(24):3823–34.

2.	Onu C, Ongeri L, Bukusi E, Cohen CR, Neylan TC, Oyaro P, et al. Interpersonal psychotherapy for depression and posttraumatic stress disorder among HIV-positive women in Kisumu, Kenya: study protocol for a randomized controlled trial. Trials. 2016 Feb 3;17:64.

3.	Opiyo E, Ongeri L, Rota G, Verdeli H, Neylan T, Meffert S. Collaborative Interpersonal Psychotherapy for HIV-Positive Women in Kenya: A Case Study From the Mental Health, HIV and Domestic Violence (MIND) Study. J Clin Psychol. 2016;72(8):779–83.

4.	Curran GM, Bauer M, Mittman B, Pyne JM, Stetler C. Effectiveness-implementation Hybrid Designs. Med Care. 2012 Mar;50(3):217–26.

5.	Jiang RF, Tong H, Delucchi KL, Neylan TC, Shi Q, Meffert SM. Interpersonal psychotherapy versus treatment as usual for PTSD and depression among Sichuan earthquake survivors: a randomized clinical trial. Conflict and Health. 2014 Sep 4;8(1):14.

6.	Weiss WM, Murray LK, Zangana GAS, Mahmooth Z, Kaysen D, Dorsey S, et al. Community-based mental health treatments for survivors of torture and militant attacks in Southern Iraq: a randomized control trial. BMC Psychiatry. 2015 Dec;15(1):249.

7.	Asrat B, Schneider M, Ambaw F, Lund C. Effectiveness of psychological treatments for depressive symptoms among people living with HIV/AIDS in low- and middle-income countries: A systematic review and meta-analysis. Journal of Affective Disorders. 2020 Jun 1;270:174–87.

8.	Singla DR, Kohrt BA, Murray LK, Anand A, Chorpita BF, Patel V. Psychological Treatments for the World: Lessons from Low- and Middle-Income Countries. Annu Rev Clin Psychol. 2017 May 8;13:149–81.

9.	StataCorp. 2017. Stata Statistical Software: Release 15. College Station, TX: StataCorp LLC.; 2017.

10.	Meffert SM, Neylan TC, Chambers DA, Verdeli H. Novel implementation research designs for scaling up global mental health care: overcoming translational challenges to address the world’s leading cause of disability. Int J Ment Health Syst. 2016;10:19.
